# Increased Body Weight and Fat Mass After Subchronic GIP Receptor Antagonist, but Not GLP-2 Receptor Antagonist, Administration in Rats

**DOI:** 10.3389/fendo.2019.00492

**Published:** 2019-08-06

**Authors:** Sara Baldassano, Lærke Smidt Gasbjerg, Hüsün Sheyma Kizilkaya, Mette Marie Rosenkilde, Jens Juul Holst, Bolette Hartmann

**Affiliations:** ^1^Department of Biomedical Sciences, University of Copenhagen, Copenhagen, Denmark; ^2^Dipartimento di Scienze e Tecnologie Biologiche Chimiche e Farmaceutiche, Università di Palermo, Palermo, Italy; ^3^Novo Nordisk Foundation Center for Basic Metabolic Research, University of Copenhagen, Copenhagen, Denmark

**Keywords:** glucose-dependent insulinotropic polypeptide (GIP), GIP receptor, GIP receptor antagonist, glucagon-like peptide-2 (GLP-2), lipid homeostasis

## Abstract

Glucose-dependent insulinotropic polypeptide (GIP) and glucagon-like peptide-2 (GLP-2) are hormones secreted from the enteroendocrine cells after a meal. They exert their actions through activation of G protein-coupled receptors (R), the GIPR and GLP-2R, respectively. Both have been reported to influence metabolism. The purpose of the study was to investigate the role of the hormones in the regulation of lipid and bone homeostasis by subchronic treatment with novel GIPR and GLP-2R antagonists. Rats were injected once daily with vehicle, GIPR, or GLP-2R antagonists for 3 weeks. Body weight, food intake, body composition, plasma lipoprotein lipase (LPL), adipokines, triglycerides and the marker of bone resorption carboxy-terminal collagen crosslinks (CTX), were examined. In rats, subchronic treatment with GIPR antagonist, rat GIP (3-30)NH_2_, did not modify food intake and bone resorption, but significantly increased body weight, body fat mass, triglycerides, LPL, and leptin levels compared with vehicle treated rats. Subchronic (Pro3)GIP (a partial GIPR agonist), GLP-2(11-33), and GLP-2(3-33) (GLP-2R antagonists) treatment did not affect any parameter. The present results would be consistent with a role for GIP, but not GLP-2, in the maintenance of lipid homeostasis in rats, while neither GIPR nor GLP-2R antagonism appeared to influence bone resorption in rats.

## Introduction

Glucose-dependent insulinotropic peptide (GIP) and glucagon-like peptide-2 (GLP-2) are peptide hormones released from K and L cells of the gastrointestinal tract, respectively (1, 2). Both exert their functions through activation of specific G protein-coupled receptors (3, 4). The GIP receptor (GIPR) is expressed on different cell type including pancreatic cells, adipocytes (5), osteoblasts (6), and osteoclasts (7). The GLP-2 receptor (GLP-2R) is widely expressed in gastrointestinal tract and central nervous system (8–10) but fewer are found in the liver, vagal afferents, and adipose tissue (11). Like the related hormone GLP-1, both GIP and GLP-2 are substrates for the ubiquitous enzyme, dipeptidyl peptidase-4 (DPP-4) which cleaves off the two N-terminal amino acids, leaving antagonistic metabolites, GIP(3-42) and GLP-2(3-33). Because of DPP-4 mediated degradation, the half-lives of GIP and GLP-2 in humans are about 7 min (12, 13).

In humans, GIP is known for post-prandial stimulation of insulin secretion, increase in adipose tissue blood flow, and stimulation of lipid deposition (14). *In vitro*, GIP stimulates adipocyte lipolysis (15) and modulates re-esterification of fat (16). However, administration of a DPP-4 resistant GIP agonist, D-Ala2-GIP, to mice fed a high fat diet (HFD), was reported to decrease lipoprotein lipase (LPL) activity and body weight (17). Furthermore, GIP administration reduced adipose tissue inflammation in DPP-4 deficient rats fed a HFD (18) and GIP-overexpression in transgenic mice reduced adipose tissue inflammation (19). On the other hand, proline-3 (Pro3)GIP, considered a GIPR antagonist, reduced visceral fat in rats (20) and the longer-acting form (Pro3)GIP[mPEG] was even more efficient than (Pro3)GIP to decrease body weight and triglyceride levels in HFD fed mice (21). We have demonstrated that (Pro3)GIP acts as a partial GIPR agonist (22) and (Pro3)GIP which was initially characterized as a GIPR antagonist (23) has also shown to act as a partial GIPR agonist (24, 25). Thus, the controversial results could be due to agonistic properties of (Pro3)GIP in rats.

GLP-2 is known for its intestinotropic effects (26). It facilitates intestinal absorption of lipids (27, 28) and enhances chylomicron secretion from the intestine (27, 29, 30), and blocking GLP-2R signaling results in increased dyslipidemia and hepatic lipid accumulation in HFD fed mice (31). It also influences glucose metabolism (32–37).

Both GIP and GLP-2 are involved in bone remodeling. Judged on lower bone resorption markers, GIP markedly inhibits bone resorption in humans (38), whereas studies in GIPR knockout (KO) mice are contradictory, showing both greater (39) and lower (40, 41) plasma levels of collagen degradation fragments that are released during osteoclastic bone resorption. GLP-2 caused a pronounced and dose-related decrease (42, 43) in the bone resorption marker carboxy-terminal collagen crosslinks (CTX), but there are no studies regarding the effects of endogenous GLP-2 on bone homeostasis.

Lipid and bone metabolism appear to be related. The adipose tissue may influence bone remodeling (44) and adipose tissue inflammation favors bone degradation (45). Thus, maintenance of normal lipid homeostasis is essential not only to prevent lipid imbalance but also to preserve bone homeostasis. The mechanisms linking fat accumulation to bone health are unclear. Therefore, we investigated some physiological actions of GIP and GLP-2 in rats by subchronic treatment with a novel GIPR antagonist, GIP(3-30)NH_2_ (14, 22), a partial agonist, (Pro3)GIP and two different GLP-2R antagonists [GLP-2(11-33), and GLP-2(3-33)] (31, 46, 47) in an attempt to (1) evaluate their action in the regulation of lipid and bone homeostasis and (2) identify potential mechanisms linking the two together.

## Materials and Methods

### Animals

All experiments were in accordance with internationally accepted principles for the care and use of laboratory animals and in compliance with an animal experiment license (2013/15–2934-00833) issued (to JH) by the Danish Committee for Animal Research. Male Wistar rats (Taconic, Denmark) weighing ~200 g at the time of arrival were housed two in each cage at the Panum Institute, Copenhagen, Denmark. They were kept in temperature- (21°C) and humidity-controlled (55%) rooms with light/dark cycles of 12 h with free access to standard rat chow and water.

### Experimental Protocol

After 1 week of acclimatization, animals were allocated into five groups (*n* = 6/each group). Rats received either vehicle, rat GIP(3-30)NH_2_, (25 nmol/kg b.w.), human (Pro3)GIP (25 nmol/kg b.w.), human GLP-2(3-33) (25 nmol/kg b.w.), or human GLP-2(11-33) (25 nmol/kg b.w.) sc daily at 6 PM for 3 weeks. Doses were chosen on the basis of the published literature (20–22, 31, 48, 49) assuming that the volume of distribution (in %) is comparable in mice and rats. To study bone resorption, blood was collected from the sublingual vein of rats at −2, −1, 0, 6, 10, 14, 17, 21 days of treatment. The day after the last injection, the rats were euthanized and blood was collected from the vena cava for further biochemical analysis. The liver was excised and samples were snap-frozen in liquid nitrogen and stored at −80°C until assayed.

### Food Intake, Body Weight, and Body Composition

Food intake was measured twice a week during the study period at 10 AM by subtracting the leftover weights from the initial weights and calculated as cage means (g/rat). Body weight was monitored weekly at 10 AM. Measures of body lean and fat mass were determined in live, conscious animals using quantitative magnetic resonance spectroscopy (EchoMRI-700TM; Echo MRI).

### Peptides

Synthetic rat GIP(3-30)NH_2_, human GLP-2(3-33) and human GLP-2(11-33) were from Caslo laboratory (Kongens Lyngby, Denmark). Human (Pro3)GIP was a generous gift from Novo Nordisk A/S, Bagsværd, Denmark. The purity (≥95%) and correctness of structures were confirmed by mass, sequence, and HPLC analysis. For injections, the peptides were dissolved in PBS buffer containing 3.5 mg/ml Hemaccel (Behringwerke AG, Marburg, Germany), which was also used for control injections. Each injection volume was 400 μl.

### Blood Samples

To obtain serum, blood samples were allowed to clot for 30 min and were then centrifuged (10 min at 3,000 × g) and stored at −20°C until analysis. To obtain plasma, blood was collected into chilled tubes containing in final concentrations EDTA 3.9 mmol/l and valine-pyrolidide 0.01 mmol/l (a DPP-4 inhibitor, a gift from Novo Nordisk A/S, Bagsvaerd, Denmark). The samples were centrifuged (10 min at 3,000 × g, 4°C) and plasma was kept at −80°C until analysis.

### Biochemical Analysis

Bone resorption was assessed by measurements of degradation product from C-terminal cross linked telopeptides of type 1 collagen (CTX, RatLaps; IDS Immunodiagnostic Systems GmbH, Frankfurt am Main, Germany). CTX values were expressed as percent of the mean of the baseline values (days−2,−1, 0) of the individual animal. The intra- and interassay coefficient of variation (CV's) of the assay is in the range 6–10 and 10–15%, respectively. Resistin was measured using rat resistin enzyme immunoassay kit (Bertinpharma, Montigny le Bretonneux, France). The intra-assay CV is <5%, the inter-assay <10%. Leptin was measured using rat leptin enzyme immunoassay kit (Mediagnost, Reutlingen, Germany). The intra- and inter-assay CV are <5%. Adiponectin was measured using rat adiponectin ELISA kit (Millipore, St. Charles, Missouri, USA). The intra- and inter-assay CV are <2 and <9%, respectively. LPL was measured using lipoprotein lipase ELISA kit (Cell Biolabs, San Diego, CA, USA). The intra- and inter-assay CV are <4 and <8%, respectively. All samples were analyzed in duplicates in the same assay to prevent inter assay variation.

### Pharmacokinetic Study

Samples were taken at 0, 5, 10, 20, 30, and 45 min post-injection of GIP(3-30)NH_2_. Blood was immediately aliquoted into tubes containing final concentrations of EDTA 3.9 mmol/l. Samples were centrifuged (3,000 × g for 10 min at 4°C) and the plasma stored at −80°C until analysis. GIP(3-30)NH_2_ concentrations were measured in plasma with an in-house-developed radioimmunoassay (50) using a polyclonal antibody (code no. 95236) raised in rabbits against porcine GIP(1-30)NH_2_. As standard we used rat GIP(3-30)NH_2_ and the tracer was ^125^I-labeled human GIP(3-30NH_2_).

### Analysis of Liver and Plasma Triglycerides

Total liver lipids were extracted as previously described (31). The extracts were evaporated under vacuum in a rotary evaporator and re-suspended in 1 ml of isopropanol for quantification of triglycerides. Plasma and intrahepatic triglycerides were assessed using triglyceride determination kit (Sigma-Aldrich, Saint Louis, USA). All samples were analyzed in duplicates in the same assay to prevent inter assay variation.

### Statistics

Results are shown as means ± SEM. The letter n indicates the number of animals. The comparison between the groups was performed by one way ANOVA followed by Tukey's post-test using Prism Version 5.0 Software (Graph Pad Software, Inc., San Diego, CA, USA). A *P* < 0.05 was considered to be statistically significant.

## Results

### Effects of GIP(3-30)NH_2_ or (Pro3)GIP Treatment in Rats

**Figure 1:**
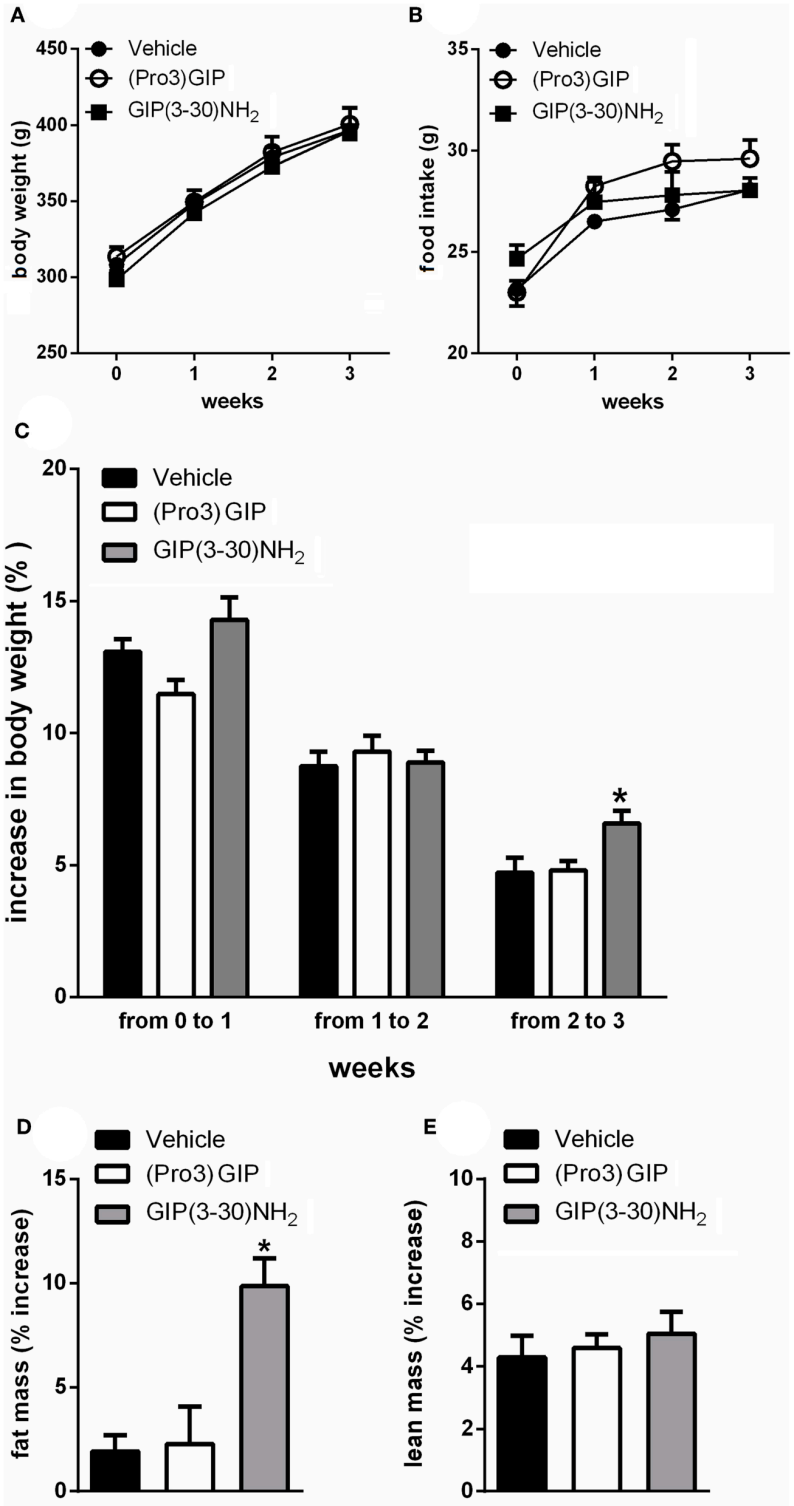
Effects of the subchronic treatment with GIP (3–30)NH_2_ (25 nmol/kg b.w.), (Pro3)GIP (25 nmol/kg b.w.) or vehicle treated on food intake, body weight, total body composition: Body weight **(A)**, Food intake **(B)**, Body weight gain **(C)**, Changes in total fat **(D)**, and lean **(E)** mass, measured by NMR. Data are mean values ± S.E.M. (*n* = 6 rats/group). *p < 0.05 vs. vehicle treatment.

It was first investigated if treatment with the GIPR antagonist, GIP(3-30)NH2 affected food intake, body weight, or body composition. In rats, 3 weeks treatment with GIP(3-30)NH2 (25 nmol/kg b.w.) had no measurable effect on body weight and food intake (Figures 1A,B). Moreover, the rats did not show differences in body weight gain during the first 2 weeks of treatment with GIP(3-30)NH2 but exhibited significantly increased body weight gain in the third week of treatment compared with vehicle treated rats (Figure 1C). Moreover, treatment with GIP(3-30)NH2 resulted in increased body fat mass measured by NMR spectroscopy at the end of the treatment period compared with vehicle treated rats (Figure 1D) while no difference in lean mass was observed (Figure 1E). Administration of the GIPR partial agonist (Pro3)GIP (25 nmol/kg b.w.) was not associated with changes on food intake, body weight or body composition during the treatment period (Figure 1).

**Figure 2:**
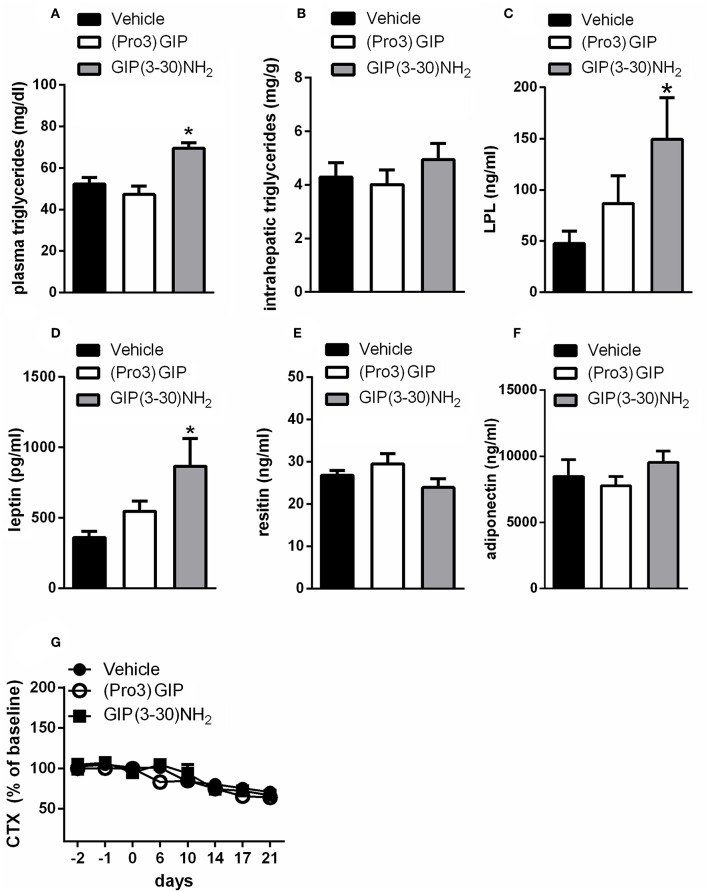
Effects of the subchronic treatment with GIP (3-30)NH_2_ (25 nmol/kg b.w.), (Pro3)GIP (25 nmol/kg b.w.) or vehicle treated on triglycerides, adipokines, and CTX: plasma triglycerides **(A)**, intrahepatic triglycerides **(B)**, plasma LPL **(C)**, plasma leptin **(D)**, plasma resistin **(E)**, plasma adiponectin **(F)**, Serum CTX levels **(G)**. Animals were bled terminally and adipokine levels were determined by ELISA as described under “materials and methods.” CTX values were expressed as percent of the mean of the baseline values (days−2,−1, 0) of the individual animal. Data are shown as mean ± SEM, n = 6 per group. *p < 0.05 vs. vehicle treatment.

Subchronic administration of GIP(3-30)NH_2_ increased plasma triglyceride levels compared with vehicle treated rats (Figure 2A) but failed to change intrahepatic lipid concentration (Figure 2B). It also significantly enhanced plasma lipase lipoprotein (LPL) levels (Figure 2C). Moreover, 3 weeks treatment with GIP(3-30)NH_2_ increased plasma leptin (Figure 2D), while no significant effect on circulating levels of resistin or adiponectin was observed (Figures 2E,F). Pharmacokinetic measurements of GIP(3-30)NH_2_ revealed that a maximum concentration of ~17 nM was achieved 10 min after administration and that the antagonist is cleared from plasma after about 120 min (data not shown).

Administration of (Pro3), that acts as a partial agonist with a Ki of 19 nM for the rat GIPR, did not modify triglycerides, intrahepatic triglyceride content, plasma LPL, or plasma levels of adipokines (Figure 2).

It was also examined whether the GIPR antagonist influences bone resorption in rats. There was a decline in CTX levels by about 30% throughout the course of the study in all the groups of rats (Figures 2G, **4F**). The treatment with GIP(3-30)NH_2_ or (Pro3)GIP did not affect serum CTX levels compared with vehicle treated rats during the treatment period (Figure 2G).

### Effects of GLP-2(11-33) or GLP-2(3-33) Treatment in Rats

**Figure 3:**
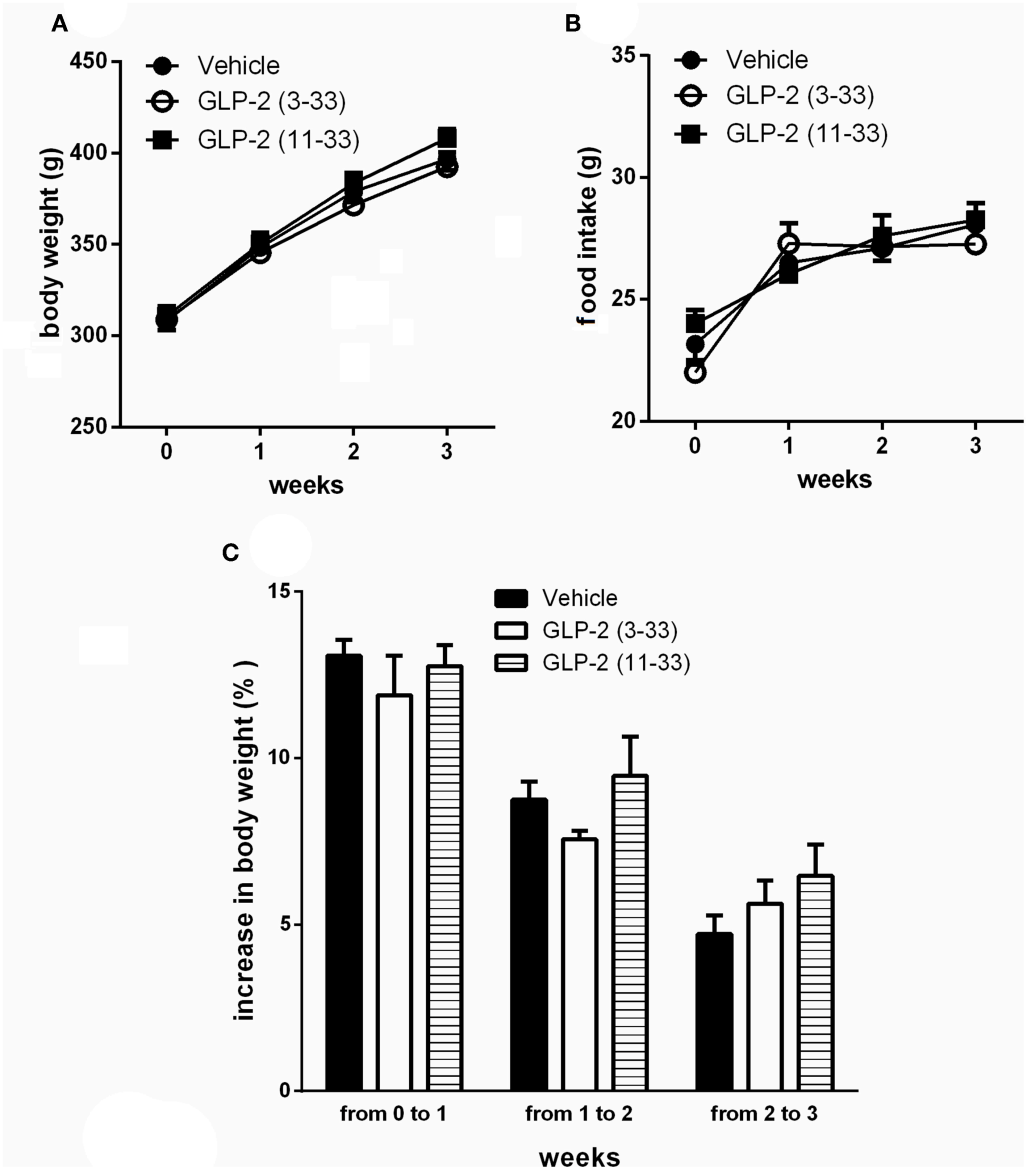
Effects of the subchronic treatment with GLP-2(11-33) (25 nmol/kg b.w.), GLP-2(3-33) (25 nmol/kg b.w.) or vehicle treated on body weight **(A)**, food intake **(B)**, and body weight gain **(C)**. Data are mean values ± S.E.M (*n* = 6 rats/group).

**Figure 4:**
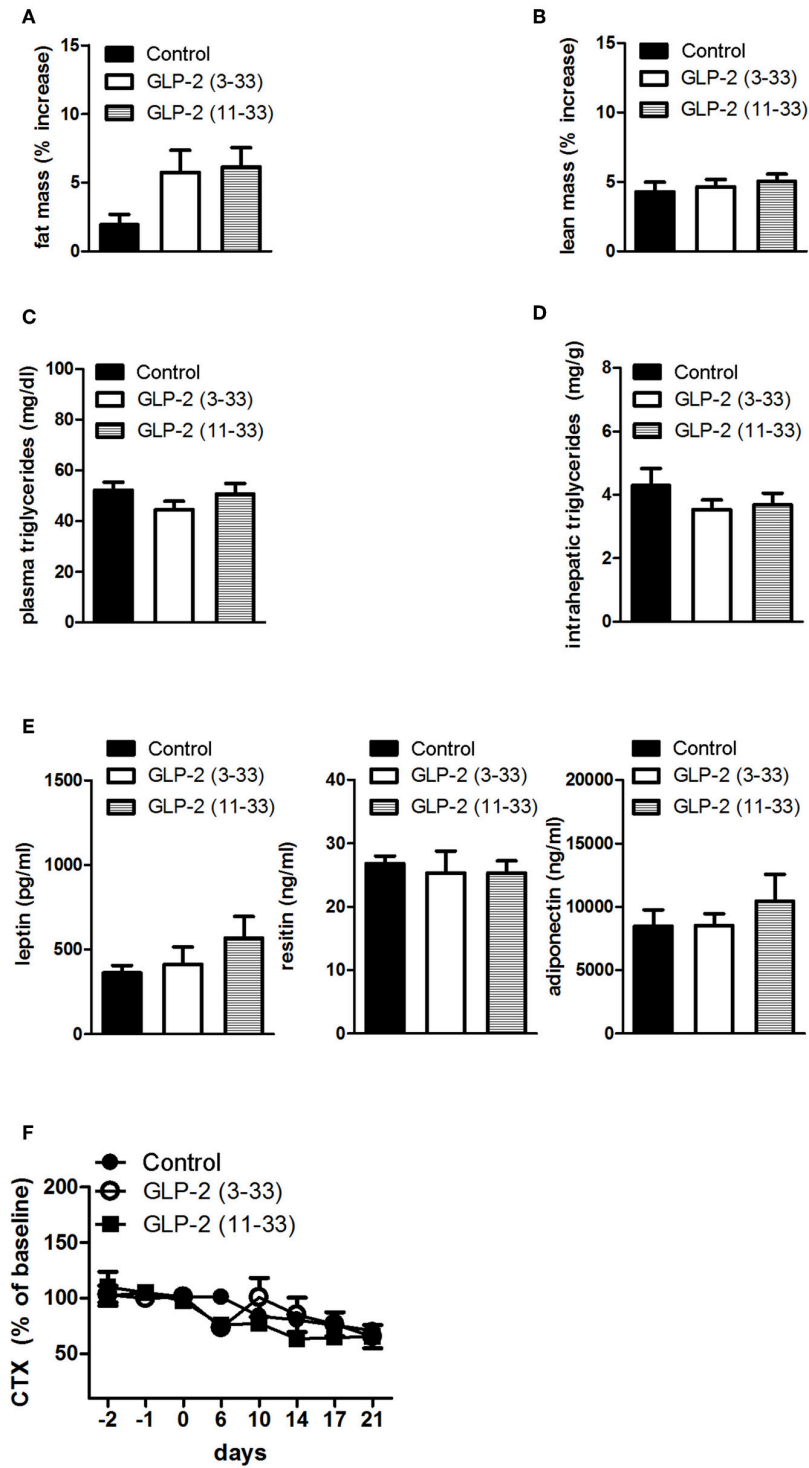
Effects of the subchronic treatment with GLP-2(11-33) (25 nmol/kg b.w.), GLP-2(3-33) (25 nmol/kg b.w.) or vehicle treated on total body composition, triglycerides, adipokines, and CTX: Changes in total fat **(A)** and lean **(B)** mass, measured by NMR, plasma triglycerides **(C)**, intrahepatic triglycerides **(D)**, plasma adipokine levels **(E)**, serum CTX levels **(F)** expressed as percent of the mean of the baseline values (days−2,−1, 0) of the individual animal. Data are mean values ± S.E.M. (*n* = 6 rats/group).

Treatment with the GLP-2R antagonists, GLP-2(11-33) (25 nmol/kg b.w.), and GLP-2(3-33) (25 nmol/kg b.w.) for 3 weeks did not modify food intake or body weight compared to vehicle treated rats (Figures 3A–C). Body mass composition (Figures 4A,B), plasma triglycerides (Figure 4C), intrahepatic triglycerides content (Figure 4D), or plasma adipokines such as leptin, resistin, and adiponectin were similar to vehicle treated rats (Figure 4E). The treatment with GLP-2(11-33) (25 nmol/kg b.w.) and GLP-2(3-33) (25 nmol/kg b.w.) did not change the serum levels of CTX during the course of the study (Figure 4F).

## Discussion

The ability of GIP to influence body weight and body composition remains unclear. Judging from the results of this study, GIP might appear to influence lipid metabolism in rats. Indeed, the treatment with a novel GIPR antagonist, GIP(3-30)NH_2_, led to increased plasma triglyceride levels and body fat mass during the third week of administration while no effects were found following GLP-2R antagonists treatment. Neither GIP nor GLP-2 appeared to be essential for the regulation of bone homeostasis in rats, although exogenous administration of both hormones has been demonstrated to reduce bone resorption in various experimental settings (6, 40–43, 48, 51).

To investigate the role of GLP-2 in lipid homeostasis we used two different potential GLP-2 R antagonists GLP-2(11-33) (37) and GLP-2(3-33) (8, 47). The rats were chronically treated once a day by s.c. injection for 3 weeks. The treatment with GLP-2(11-33) or GLP-2(3-33) did not modify body weight or composition, food intake, biochemical parameters related with lipid metabolism such as plasma triglycerides, plasma adipokines, and intrahepatic lipid.

The extent of GLP-2R antagonism elicited by our treatment is not clear. The antagonists are relatively weak (52) and their half-life after s.c. administration in rats is unknown but must be very short amounting to at the most an hour. Therefore, antagonism has only been present for a very short period, and this could influence the results of the experiment. In future studies, it will be necessary to investigate the doses of the antagonists required to block the actions of physiological levels of GLP-2 *in vivo*. However, these negative GLP-2 data could help researchers, with further study, to give a fuller account of GLP-2 action.

To study the action of endogenous GIP in lipid metabolism, we used a novel GIPR antagonist, GIP(3-30)NH_2_ (22, 53) and a well-described GIPR partial agonist, (Pro3)GIP (25). GIP(3-30)NH_2_ acts as specific and efficacious GIPR antagonist in humans (14, 54). It inhibits the insulinotropic effects of GIP and antagonizes the liporegulatory and vasodilatory effects of GIP (55). In our rats, the chronic treatment with GIP(3-30)NH_2_ induced body weight gain and significantly increased total body fat mass. In order to investigate whether the variation in body weight and consequently in the body composition was due to changes in food intake, this was measured on different days through the course of the study. However, the food intake was unchanged during the 3 weeks of treatment. Therefore, we investigated the relationship of the increase in body fat mass to changes in lipid homeostasis. First, plasma triglycerides were measured. The treatment with GIP(3-30)NH_2_ in rats significantly increased plasma triglyceride concentration, as is often seen in obesity. Thereafter, we looked into possible mechanisms whereby endogenous GIP might influence the fat deposition in the adipose tissue. Triglyceride levels are regulated by LPL, an enzyme that hydrolyzes triglycerides and releases free fatty acids and monoglycerides that are stored in adipose tissue (56). GIP(3-30)NH_2_ treatment significantly increased LPL levels compared with vehicle treated rats, suggesting that blocking the GIPR signaling promotes the triglyceride accumulation in the adipose tissue by increasing circulating LPL concentration. In fact, manipulation of LPL expression causes imbalances in the partitioning of fatty acids among peripheral tissues, which have major effects on lipid and glucose metabolism (57–59). Our results are consistent with the finding that chronic administration of the long acting GIP agonist, D-Ala 2-GIP, in HFD fed mice decreased LPL activity and body weight (17). Moreover, GIP-overexpression in transgenic mice improved systemic metabolic phenotype and reduced adipose tissue inflammation (19). Previously, studies have shown that GIP stimulates adipocytes lipolysis on adipocytes cell line (15) and modulates reesterification of lipids (16). In contrast, GIP stimulates LPL activity leading to triglyceride accumulation in cells (60, 61). In particular, GIP was shown to increase LPL enzyme activity, in an insulin-dependent manner, in cultured 3T3-L1 adipocytes, rodent adipocytes, and subcutaneous human (61–64). These contrasting results suggest that the *in vitro* studies on cell lines cannot be translated to adipose tissue effects *in vivo*.

The liver is central for maintenance of lipid homeostasis (65). Thus, we also looked at the effects of the 3 weeks of treatment with GIP(3-30)NH_2_ on liver lipid accumulation in rats. We did not observe differences in the intrahepatic triglyceride content in the GIP(3-30)NH_2_ treated group compared with vehicle treated group suggesting that antagonism of GIPR signaling did not promote the triglyceride accumulation in the liver.

Adipose tissue secretes bioactive peptides named “adipokines” which act locally and distantly through autocrine, paracrine, and endocrine effects (66). Previous studies in transgenic mouse models indicated that GIP may modulate the adipokine profile secreted from the adipose tissue (67, 68). In our study, GIP(3-30)NH_2_ treatment did not affect resistin and adiponectin levels but significantly increased leptin levels consistent with the expansion of the white adipose tissue.

Our data may suggest that the GIP system is basically different in humans and rodents with regards to adipose tissue. In humans, GIP appears to act as a lipid storage hormone. It increases adipose tissue blood flow and stimulates fat deposition (69, 70) and the GIPR antagonist, human GIP(3-30)NH_2_, decreases adipose tissue triacylglyceride uptake and increases the free fatty acid/glycerol ratio (14, 55). In rodents, GIP stimulates lipolysis (15), decreased lipase lipoprotein (LPL) activity and body weight (17), adipose tissue inflammation (18, 19), visceral fat (20), and triglyceride levels (21). The GIP antagonist, rat GIP(3-30)NH_2_, increases body weight, total body fat mass, leptin, and LPL in rats. Thus, it is becoming clear that important differences in metabolic and signal transduction may exist between rodent and human adipose tissue. Thus, caution should be exercised when extrapolating information from one species to another.

In our experiments, the treatment with the partial GIP agonist, (Pro3)GIP did not modify any of the investigated parameters in rats. This is in agreement with a previous study by Irwin et al. showing no effect of (Pro3)GIP on food intake, body weight, insulin concentrations, and islet morphology (71). Previously, using the same dose, (Pro3)GIP was shown to be able to reduced body weight, plasma insulin, and triglyceride levels in HFD fed mice (21). Likely, the discrepancies could be due to the type of diet. In fact, McClean et al. tested the (Pro3)GIP effects in HFD fed mice, a diet that induces metabolic syndrome (72). Also during catch-up growth in female rats, associated with metabolic syndrome, the administration of (Pro3)GIP reduced visceral fat mass and adipocyte hypertrophy without variations in body weight. However, we previously showed lower activities of human, mouse, and rat (Pro3)GIP in the rodent GIP systems compared with the human system (25) highlighting the significant interspecies difference within the GIP system.

Adipose tissue and bone are closely related. Previous studies have widely linked lipid intake and inflammation status, key protagonists involved in bone resorption (73, 74). Inflammation favors bone degradation by stimulating osteoclast activity while inhibiting osteoblast-related bone formation, which leads to unbalanced bone remodeling and subsequent bone loss. Thus, we decided to investigate the influence of the endogenous GIP and GLP-2 in bone resorption by measuring serum CTX levels. We observed a decline in CTX levels throughout the course of the study in all the groups of rats, including control treated group. A factor hampering the measurement of bone markers in rats is a continuous decline of some markers (75). CTX levels decreases with the age in rats (76) and our data suggests that CTX decline is already evident within 3 weeks.

The results of the pharmacokinetic study of GIP(3-30)NH_2_ showed an average peak level of ~17.000 pmol/l and revealed that the half-life after injection was ~20 min. This means that exposure for the antagonist was provided for maximally 2 h. In addition, previous *in vitro* experiments (22) would suggest that efficient antagonism requires a considerable excess of antagonist compared to the levels of the endogenous hormones, which further reduces the time of efficient antagonism. As for GLP-2, it can be concluded that the animals have been without GIPR antagonism for most of the time during the 3 weeks. This raises the question of the mechanism behind the observed changes in lipid metabolism. It seems unlikely that antagonism for just a small fraction of the time could result in major changes in body weight and lipid metabolism as observed. Rather it could be suggested that a brief but extensive disturbance of the effects of GIP could lead to a compensatory response of a longer duration, which in turn could have consequences for lipid metabolism. Further experiments, involving repeated administration of the antagonists and measurements of possible compensatory mechanisms (e.g., increases in GIP levels after antagonist administration) as well as changes in glucose and insulin levels are required to elucidate this.

We were unable to detect increases in food intake in the animals treated with the GIP antagonist, although an increase in body weight was observed. Probably, small increases in food intake, not registered by our measurements, explain the changes not only in body weight, but also in triglyceride levels. Alternatively, it has been reported that GIP might increase lipid oxidation in rodents (77). Conversely, GIPR antagonism might decrease lipid oxidation, which might lead to increased circulating and tissue triglyceride levels. To maintain sufficient energy metabolism this would imply an increase in non-lipid oxidation, which would be expected to translate into increased food intake, which was not observed. It is also possible that a reduction in physical activity and energy expenditure could explain the changes in lipid metabolism, however, we did not monitor locomotor activity so this is only speculative. Thus, it is difficult to fully explain the mechanism of action of the GIPR antagonists with respect to fat mass and body weight.

The subchronic treatment with GIP(3-30)NH_2_, (Pro3)GIP and the GLP-2R antagonists, GLP-2(11-33), and GLP-2(3-33), did not affect CTX levels compared with control treated-rats which suggests that endogenous GIP and GLP-2 do not influence bone resorption in normal rats. However, because of the short duration of the antagonism the negative findings cannot be assigned much weight. Thus, GLP-2 has been shown to suppress bone resorption in humans (41, 78, 79) and GIP administration increase bone density in ovariectomized rats (48) while GIP receptor knockout mice have decreased bone size and mass, altered bone microstructure, and turnover (80). Mice with overexpression of GIP had increased markers of bone formation, decreased markers of bone resorption, and increased bone mass (81). Moreover, acute administration of GIP in humans inhibited bone resorption (38). In addition, we cannot exclude that the decline in CTX levels that we observed during the course of study in all groups contributed to mask any changes in marker levels caused by the different treatments.

In conclusion, treatment with GIP(3-30)NH_2_ antagonist affected lipid metabolism in rats whereas a partial GIPR agonist or a GLP-2R antagonists appeared not to influence neither lipid metabolism nor bone resorption in rats.

## Data Availability

All datasets generated for this study are included in the manuscript and/or the supplementary files.

## Ethics Statement

All experiments were in accordance with internationally accepted principles for the care and use of laboratory animals and in compliance with an animal experiment license (2013/15–2934-00833) issued (to JH) by the Danish Committee for Animal Research.

## Author Contributions

SB and BH concepted and designed the study, performed the experiments, interpreted data, revised the article, and approved the final version. HK performed experiments. LG, HK, MR, and JH contributed with interpretation of data, article revision, final approval, and agreement.

### Conflict of Interest Statement

The authors declare that the research was conducted in the absence of any commercial or financial relationships that could be construed as a potential conflict of interest.
